# Phenotypic convergence in a natural *Daphnia* population acclimated to low temperature

**DOI:** 10.1002/ece3.8217

**Published:** 2021-10-12

**Authors:** Christian Werner, Kathrin A. Otte, Eric von Elert

**Affiliations:** ^1^ Aquatic Chemical Ecology Institute for Zoology University of Cologne Köln Germany

**Keywords:** *Daphnia*, intraspecific competition, membrane fluidity, polyunsaturated fatty acid, PUFA

## Abstract

Fluidity of a given membrane decreases at lower ambient temperatures, whereas it rises at increasing temperatures, which is achieved through changes in membrane lipid composition. In consistence with homeoviscous adaptation theory, lower temperatures result in increased tissue concentrations of polyunsaturated fatty acids (PUFAs) in *Daphnia magna*, suggesting a higher PUFA requirement at lower temperatures. However, so far homeoviscous adaptation has been suggested for single or geographically separated *Daphnia* genotypes only. Here, we investigated changes in relative fatty acid (FA) tissue concentrations in response to a lower temperature (15°C) within a *D. magna* population. We determined juvenile growth rates (JGR) and FA patterns of 14 genotypes that were grown on *Chlamydomonas klinobasis* at 15°C and 20°C. We report significant differences of JGR and the relative body content of various FAs between genotypes at either temperature and between temperatures. Based on slopes of reaction norms, we found genotype‐specific changes in FA profiles between temperatures suggesting that genotypes have different strategies to cope with changing temperatures. In a hierarchical clustering analysis, we grouped genotypes according to differences in direction and magnitude of changes in relative FA content, which resulted in three clusters of genotypes following different patterns of changes in FA composition. These patterns suggest a lower importance of the PUFA eicosapentaenoic acid (EPA, C20:5ω3) than previously assumed. We calculated an unsaturation index (UI) as a proxy for membrane fluidity at 15°C, and we neither found significant differences for this UI nor for fitness, measured as JGR, between the three genotype clusters. We conclude that these three genotype clusters represent different physiological solutions to temperature changes by altering the relative share of different FAs, but that their phenotypes converge with respect to membrane fluidity and JGR. These clusters will be subjected to different degrees of PUFA limitation when sharing the same diet.

## Graphical abstract

In consistence with the homeoviscous adaptation theory, the concept of maintaining a constant membrane fluidity at changing temperatures, it has been shown for single *D. magna*, genotypes that lower temperatures result in increased tissue concentrations of PUFAs, suggesting a higher PUFA requirement at lower temperatures. Here, we investigated homeoviscous adaptation to a lower temperature (15°C) within a *D. magna* population by calculating the change of the relative content of individual fatty acids between 15°C and 20°C for 14 genotypes of a natural *D. magna* population. We applied a hierarchical clustering analysis that grouped genotypes according to differences in their fatty acid alteration profiles, which resulted in three different clusters of genotypes, that differ in terms of changes in their fatty acid content but whose phenotypes converge in terms of an unsaturation index and juvenile somatic growth, which were determined for each genotype as well.

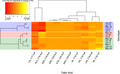

## 1 INTRODUCTION

The freshwater crustacean *Daphnia* occupies a keystone position in pelagic food webs by being the primary conveyor of energy and biomass as well as limiting biochemicals from primary producers, the phytoplankton, to higher trophic levels of consumers like planktivorous fish (Gaedke & Straile, 1998; Taipale et al., 2016).

The nutritional value and thus the quality of the diet of *Daphnia* is determined by various parameters, including toxicity, digestibility, mineral content, and biochemical composition of the phytoplankton, the latter two of which have been the subject of several studies in the last decades (Ahlgren et al., 1990; Becker & Boersma, 2003; Boersma et al., 2008; Müller‐Navarra, 1995a; Park et al., 2002; Sundbom & Vrede, 1997). In addition to the stoichiometric ratio of carbon to nutrients in the diet (Ravet & Brett, 2006; Sterner et al., 1993; Urabe et al., 1997), the performance of *Daphnia* has often been linked to the availability of dietary polyunsaturated fatty acids (PUFAs), that is, fatty acids with two or more double bonds in their carbon chain (von Elert, 2002; Müller‐Navarra, 1995b; Wacker & von Elert, 2001).

In poikilotherms such as *Daphnia*, PUFAs are important cell membrane components that play a significant role in homeoviscous adaptation (HVA, Hazel, 1995), i.e. an adjustment of membrane lipid composition to maintain proper membrane fluidity and function at different temperatures. In general, the fluidity of a given membrane is reduced at decreasing ambient temperatures, whereas it is increased at increasing temperatures (Hazel, 1995). Furthermore, PUFAs may serve as precursors for the biosynthesis of eicosanoids and immune functions in *Daphnia* (Schlotz et al., 2012, 2016). However, the presence and amount of PUFAs in the diet vary drastically between different phytoplankton groups (Ahlgren et al., 1990; Lang et al., 2011). PUFAs are essential for invertebrates, including *Daphnia*, because they cannot be synthesized de novo by these animal groups (Beenakkers et al., 1985; Leonard et al., 2004; Stanley‐Samuelson et al., 1987). Therefore, PUFAs are either converted from other PUFAs or assimilated directly from the diet (Goulden & Place, 1993; Hulbert et al., 2005). Although previous studies suggest that at least some *Daphnia* species are able to convert ω3‐ and ω6‐PUFAs within the respective PUFA family by elongation and desaturation of PUFAs with shorter carbon chain length, the rate of this conversion does not cover the physiological requirement (von Elert, 2002; Taipale et al., 2011; Weers et al., 1997).

Several field studies provided evidence for a limitation of *Daphnia* growth when feeding on natural phytoplankton using juvenile growth rate (JGR) as a good proxy for *Daphnia* fitness (Lampert & Trubetskova, 1996): In studies that covered the whole spring and summer seasons, juvenile growth of *Daphnia* spp., a major consumer of phytoplankton, correlated highly with the dietary availability of sestonic ω3‐ and ω6‐PUFAs, particularly α‐linolenic acid (ALA, 18:3ω3; Wacker & von Elert, 2001) and eicosapentaenoic acid (EPA, 20:5ω3; Müller‐Navarra, 1995b). Although the identification of a single limiting PUFA based on correlations was not straightforward due to strong intercorrelations between different PUFAs, it was suggested that PUFA‐limitation of *Daphnia* in nature is widespread (Müller‐Navarra, 1995b; Müller‐Navarra et al., 2000; Wacker & von Elert, 2001).

Several laboratory studies supported the assumption that dietary availability of PUFAs may limit *Daphnia* fitness: In a study with *Daphnia galeata*, von Elert (2002) supplemented algal diets with single ω3‐PUFAs and showed increased juvenile growth rates when EPA, ALA, and docosahexaenoic acid (DHA, 22:6ω3) were supplemented. For EPA in particular, low dietary availability has been shown to limit the juvenile growth rate of *Daphnia* (Becker & Boersma, 2003; Sperfeld & Wacker, 2012; Windisch & Fink, 2018). Furthermore, both, reproduction (Becker & Boersma, 2005; Martin‐Creuzburg et al., 2008, 2010; Ravet et al., 2003) and population growth (Martin‐Creuzburg et al., 2010), were limited by dietary EPA availability. Consequently, EPA was found to be a PUFA whose low availability in the diet limits the fitness of different *Daphnia* species. In a study using *Daphnia pulex* and *Daphnia magna*, Ilić et al. (2019) reported equal importance of the ω6‐PUFA arachidonic acid (ARA, 20:4ω6) and of the ω3‐PUFA EPA for *Daphnia* fitness (growth and reproduction).

Not only the availability of PUFAs in the diet but also ambient temperature affects the degree of PUFA limitation of *Daphnia*: Supplementation of the two green algae *Acutodesmus obliquus* (Sperfeld & Wacker, 2012) and *Chlamydomonas klinobasis* (von Elert & Fink, 2018) with EPA increased juvenile growth rates of *Daphnia magna* at 15°C but not at 20°C, which pointed at elevated EPA requirements at the lower temperature. Accordingly, population growth rates of *Daphnia magna* grown on a PUFA‐free diet were limited by low PUFA availability at low temperatures (10°C) but not at higher temperatures (Martin‐Creuzburg et al., 2012).

In addition, it has been shown that higher concentrations of PUFAs, especially EPA, are incorporated into body tissues at lower temperatures (Isanta Navarro et al., 2019; Martin‐Creuzburg et al., 2012; Sperfeld & Wacker, 2011, 2012), which was attributed to homeoviscous adaptation (HVA), and thus, a higher PUFA requirement at lower temperatures was suggested. It appears plausible that an increased share of EPA and other PUFAs in *Daphnia* maintains membrane fluidity at low temperatures and thus ensures proper cell function (Farkas, 1979; Hazel, 1995; Werner et al., 2019), because the numerous double bonds of PUFAs result in highly bent carbon chains and thus reduced van der Waals interactions between membrane fatty acids (Murray et al., 2010). These reduced van der Waals interactions between fatty acids lead to a lower melting point of the membrane and thus to enhanced membrane fluidity at low temperatures.

For *D. magna*, lower temperatures have been shown to result in increased tissue concentrations of PUFAs, suggesting a higher PUFA requirement at lower temperatures (Sperfeld & Wacker, 2012; von Elert & Fink, 2018). These changes are consistent with the homeoviscous adaptation of poikilotherms to lower temperatures, which attempt to maintain constant membrane fluidity through changes in membrane lipid composition (Hazel, 1995). So far patterns of membrane fatty acid composition changes have been demonstrated for single or geographically separated *Daphnia* genotypes only (Coggins et al., 2021; Hahn & von Elert, 2020; Martin‐Creuzburg et al., 2019; Sperfeld & Wacker, 2012; von Elert & Fink, 2018). If these results of increased PUFA requirement would hold for all *Daphnia* genotypes in a population, and if all coexisting genotypes would respond with increased tissue concentrations of the same PUFAs, then these PUFAs might constitute a resource that potentially affects intraspecific competition. Alternatively, *Daphnia* populations may harbor standing clonal variation with respect to these changes in fatty acid composition. Accordingly, we here investigate if, upon exposure to 20°C and 15°C, *D. magna* genotypes from a single population show similar changes in membrane fluidity (estimated by calculation of an unsaturation index) and in how far these effects on membrane fluidity can be attributed to similar changes in fatty acid composition. We hypothesize to find genotype‐specific changes in fatty acid composition and further expect that these are also reflected in genotype‐specific fatty acid unsaturation as a measure of membrane fluidity.

More specifically, we used *D. magna* genotypes collected from a single summer population (May–July) of a Swedish lake. In earlier studies, the different genotypes have been distinguished using microsatellite analysis and were shown to differ in heat tolerance (Schwarzenberger et al., 2013; Werner et al., 2019). In addition, variation in juvenile growth rate and PUFA and EPA body content has been reported from growth experiments at 15 °C, a temperature assumed to constrain *Daphnia* growth (Werner et al., 2019). We examined juvenile growth rates (JGR) and the relative fatty acid composition at 15°C and 20°C for the different *D. magna* genotypes in order to assess their intrapopulation differences within and between temperatures. We used the slope of reaction norms to track changes in response to the two different temperatures. Such slopes for changes in fatty acid body content were analyzed by a linear regression model and a hierarchical clustering analysis, sorting genotypes according to similarities or differences in those changes. We tested for variation in membrane fluidity, calculated as an unsaturation index, between the different clusters of genotypes.

## 2 METHODS

### 2.1 Test species and cultures

Fourteen clonal lineages (distinguished by microsatellites) of *Daphnia magna* Straus from a single population of Lake Bysjön (located in southern Sweden: N 55.675399 E 13.545805) were examined in this study. The animals were collected as live specimens from a depth of 1–2 m with a plankton net (200 µm) during May–July 2010 (clonal lines carry names signifying the month and sequence—not date—of the original sampling, e.g., Jun 6) and grown in clonal lines (Schwarzenberger et al., 2013). *D. magna* clonal lineages were cultured in aged, aerated, and membrane‐filtered (pore size: 0.45 μm) tap water at 20 °C and a 16:8 h light:dark cycle for several generations.

Under standard conditions, animals were maintained at a maximum density of 15 individuals L^−1^ and under nonlimiting food concentrations by receiving 2 mg particulate organic carbon (POC) L^−1^ of the green alga *Chlamydomonas klinobasis*, strain 56, culture collection of the Limnological Institute at the University of Konstanz, every other day. *C. klinobasis* was grown in 5 L semicontinuous batch cultures (20°C; light intensity 120 µmol photons m^−2^ s^−1^ PAR) and a dilution rate of 0.1 d^−1^ with fresh, sterile Cyano medium (von Elert & Jüttner, 1997) containing vitamins (thiamine hydrochloride 300 nM, biotin 2 nM, and cyanocobalamine–vitamin B12 0.4 nM). This green alga contains phytosterols and C_18_‐PUFAs such as LIN (18:2ω6) and ALA (18:3ω3), but lacks C_20_‐PUFAs such as ARA (20:4ω6) and EPA (20:5ω3; Martin‐Creuzburg et al., 2005; von Elert & Fink, 2018; von Elert & Stampfl, 2000; Windisch & Fink, 2018). The test animals for the experiments performed in this study originated from mothers that had been raised under standard conditions (saturating concentrations of *C. klinobasis*) for at least five generations.

### 2.2 Growth experiments

Growth experiments at 20°C were initiated with seven to eight neonates (< 16‐h‐old) from the third clutch of clonal mothers of the different *D. magna* genotypes maintained under standard conditions at 20°C. For experiments at 15°C, clonal mothers, which were kept under standard conditions at 20°C, were transferred to 15°C once their first clutch of eggs became visible. The growth experiment itself was initiated with nine to twelve neonates (< 16‐h‐old) from the third clutch of these mothers. From then on, experiments were identically at both temperatures and carried out in triplicate with each clonal lineage. Neonates of the third clutch were placed in glass beakers filled with 250 ml of aged, aerated, and filtered (0.45 µm pore size) tap water. At the same time, subsamples of two times ten individuals were taken for the determination of the initial dry mass (*W_0_*) at the start of the experiments. The neonates were fed *C. klinobasis* in nonlimiting concentrations of 2 mg POC L^−1^ and transferred to fresh food suspensions every other day until they produced their first clutch of eggs. Then, two individuals per replicate were removed for dry mass determination, and the remaining three to four individuals were taken for fatty acid analysis.

The juvenile somatic growth rate *g* (d^−1^) was calculated according to the formula:

$$g\;=\frac{\ln\left(W_{t}\right)‐{\ln(W}_{0})}{t}$$

where *W_0_* is the initial dry mass of neonates, *W_t_* is the dry mass of individuals at the end of the experiment, and *t* is the duration of the experiment in days (Wacker & von Elert, 2001).

### 2.3 Fatty acid analysis

Fatty acids from three to four *D. magna* individuals were extracted with 5 ml dichloromethane/methanol (2:1, v:v) to determine fatty acid concentrations in the animals. As an internal standard, 5 µg of tricosanoic acid methyl ester (C23:0 ME) in isohexane was added to the samples before the further extraction steps. The extraction solvent was evaporated under a stream of nitrogen at 40°C, and the evaporated samples were then transesterified with 5 ml of 3 N methanolic HCl at 70°C for 20 min to obtain fatty acid methyl esters (FAMEs). FAMEs were extracted by adding approximately 2 ml of isohexane twice to the samples, vortexing, and then collecting the hexane layer after each of the two isohexane extraction steps. Both extracts were pooled and then evaporated to dryness under a stream of nitrogen at 40°C. The samples were then redissolved in 100 µl of isohexane per sample, of which 1 µl was injected splitlessly into a 6890‐N GC System (Agilent Technologies, Waldbronn, Germany) equipped with a DB‐225 capillary column (30 m, 0.25 mm i.d., 0.25 µm film thickness, J&W Scientific). Instrument settings were as follows: injector and FID temperatures 220°C; initial oven temperature 60°C for 1 min, followed by a 120°C/min temperature ramp to 180°C, then a ramp of 50°C/min to 200°C followed by 10.5 min at 200°C, followed by a ramp of 120°C/min to 220°C. Helium with a flow rate of 1.5 ml/min was used as the carrier gas. FAMEs were identified by comparing retention times with those of the reference compounds and then quantified using the internal standard and previously established calibration functions for each individual FAME (von Elert, 2002). To calculate relative abundances, the mass concentrations of each fatty acid were related to the total fatty acid abundance. To characterize the degree of fatty acid unsaturation in samples, we calculated the unsaturation index (UI) as (1/dw)∑A_i_u_i_, where dw is the dry weight of the sample (mg), A_i_ is the abundance (µM), and u_i_ is the unsaturation level (number of double bonds) of the *i*th fatty acid according to Martin‐Creuzburg et al. (2019). Mass concentrations of fatty acids were therefore translated into molar concentrations.

### 2.4 Statistical analysis

Data analysis was conducted using the software R (version 4.0.4). Concerning juvenile somatic growth rates (JGR) and fatty acid concentrations, mean values of all analyzed animals per jar were calculated for further analysis. The resulting datasets were analyzed by applying linear regression models to the response variables JGR and each of ten unsaturated fatty acids with genotype and temperature as predicting variables to identify differences between temperatures and between genotypes within a temperature. Normal distribution of residuals and homogeneity of variance were checked, and data were log‐transformed before applying the linear regression model if the assumptions were violated. Linear correlations of the mean relative FA data with mean JGR within both temperatures were calculated to identify significant correlations. When multiple comparisons were performed, *p*‐values were adjusted using the Benjamini–Hochberg (BH) correction (Benjamini & Hochberg, 1995).

Reaction norm slopes as a measure of change in relative fatty acid content between the two temperatures were calculated for all single fatty acids per genotype. Additionally, reaction norm slopes as a measure of change in JGR between the two temperatures were calculated per genotype. A linear regression model with genotype and fatty acid as predicting variables was applied to compare reaction norm slopes of single fatty acids among the 14 genotypes and to identify genotype‐specific profiles of fatty acid content changes. *p*‐values were adjusted using the Tukey method. Prior to the analysis, we performed a Box–Cox transformation on the data to achieve normal distribution of residuals and homogeneity of variance.

We then performed an unsupervised hierarchical clustering analysis to organize differing profiles of fatty acid content changes of the genotypes into a meaningful structure using the package dendextend (version 1.14.0). The clustering analysis organizes genotypes according to the similarity or dissimilarity of their fatty acid reaction norm slope profiles, placing the genotypes with similar profiles together as neighboring rows in a dendrogram. The relationship between genotypes and the magnitude and direction of changes in fatty acid content between temperatures is depicted graphically as a heatmap in which the rows are ordered based on the order of the hierarchical clustering dendrogram using the package gplots (version 3.1.1). Mean reaction norm slope data per fatty acid and genotype were used for the clustering analysis. We performed one‐way ANOVAs to test for differences in unsaturation index, JGR at 15°C and 20°C and changes in JGR between both temperatures among three clusters of genotypes created by the clustering analysis.

Except for the heatmap, all data were visualized using the package ggplot2 (version 3.3.3).

## 3 RESULTS

### 3.1 Temperature‐dependent growth and fatty acid content of 14 *D. magna* genotypes

**FIGURE 1:**
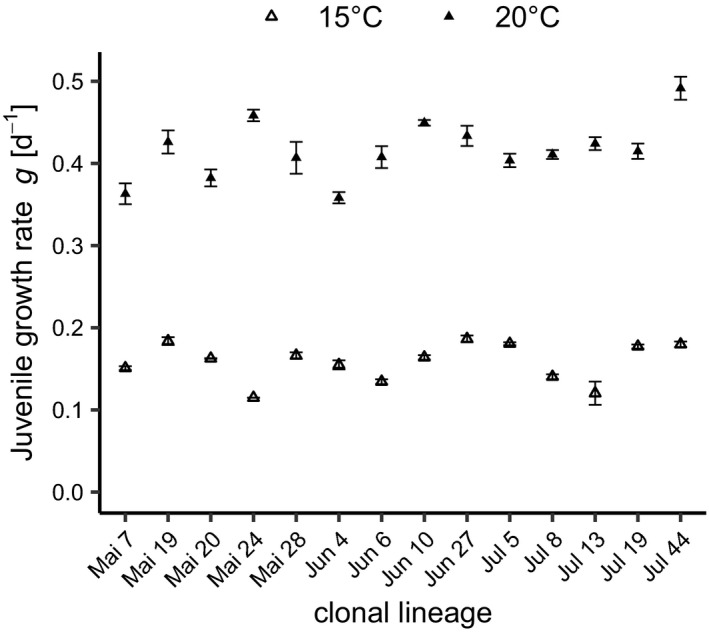
Mean (± SE) juvenile growth rates (JGR) of 14 *D. magna* clonal lineages isolated from Lake Bysjön grown at 15 and 20°C. *N* = 3; except for clonal lineages Mai 19, Mai 20 and Mai 24 at 15°C (*n* = 2)

We found intraspecific differences in the juvenile growth rates (JGR) among the 14 *D. magna* genotypes isolated from Lake Bysjön, Sweden (Table 1; Figure 1). A linear regression model analyzing the effect of temperature and genotype, and the interaction of these parameters on juvenile growth rate (JGR) revealed significant differences for the main effects as well as a significant interaction term (Table 1). Mean values of JGR were ranging from 0.11 to 0.18 day^−1^ at 15°C and from 0.36 to 0.49 day^−1^ at 20°C with animals at 20°C showing higher JGR compared to animals at 15°C (Table 1, Figure 1).

**TABLE 1:** Results from linear regression models showing effects of the factors *Genotype*, *Temperature*, and their interaction on the juvenile growth rate and the relative content of individual fatty acids of the investigated *Daphnia magna* population (all 14 genotypes included except for ARA and EPA, which were not detected in all genotypes)

Response variable	Effect	*df*	*F*	*p*
Juvenile growth rate	Genotype	13	18.89	**<.001**
	Temperature	1	5157.92	**<.001**
	Genotype × Temperature	13	10.56	**<.001**
16:1ω7 (PA)^a^	Genotype	13	1.81	.078
	Temperature	1	23.09	**<.001**
	Genotype × Temperature	13	2.00	.**049**
17:1ω9 (CA)	Genotype	13	2.67	.**009**
	Temperature	1	162.42	**<.001**
	Genotype × Temperature	13	2.15	.**036**
18:1ω9 (OA)	Genotype	13	1.61	.122
	Temperature	1	3.98	.062
	Genotype × Temperature	13	2.96	.**005**
18:1ω7 (VA)	Genotype	13	12.81	**<.001**
	Temperature	1	0.18	.67
	Genotype × Temperature	13	14.29	**<.001**
18:2ω6 (LIN)	Genotype	13	13.43	**<.001**
	Temperature	1	761.99	**<.001**
	Genotype × Temperature	13	10.55	**<.001**
18:3ω3 (ALA)	Genotype	13	4.27	**<.001**
	Temperature	1	4.52	.**049**
	Genotype × Temperature	13	2.46	.**016**
18:4ω3 (SDA)	Genotype	13	11.54	**<.001**
	Temperature	1	429.79	**<.001**
	Genotype × Temperature	13	6.77	**<.001**
20:4ω6 (ARA)^a^	Genotype	9	9.12	**<.001**
	Temperature	1	16.28	.**002**
	Genotype × Temperature			
20:3ω3 (ETE)^a^	Genotype	13	2.03	.271
	Temperature	1	72.87	**<.001**
	Genotype × Temperature	13	3.75	.**009**
20:5ω3 (EPA)^a^	Genotype	9	18.89	**<.001**
	Temperature	1	25.21	**<.001**
	Genotype × Temperature	2	2.84	.096

Note

*p*‐values in bold indicate significant effects after Benjamini–Hochberg (BH) correction.

^a^ Mark response variables that were log‐transformed before the analysis.

**FIGURE 2:**
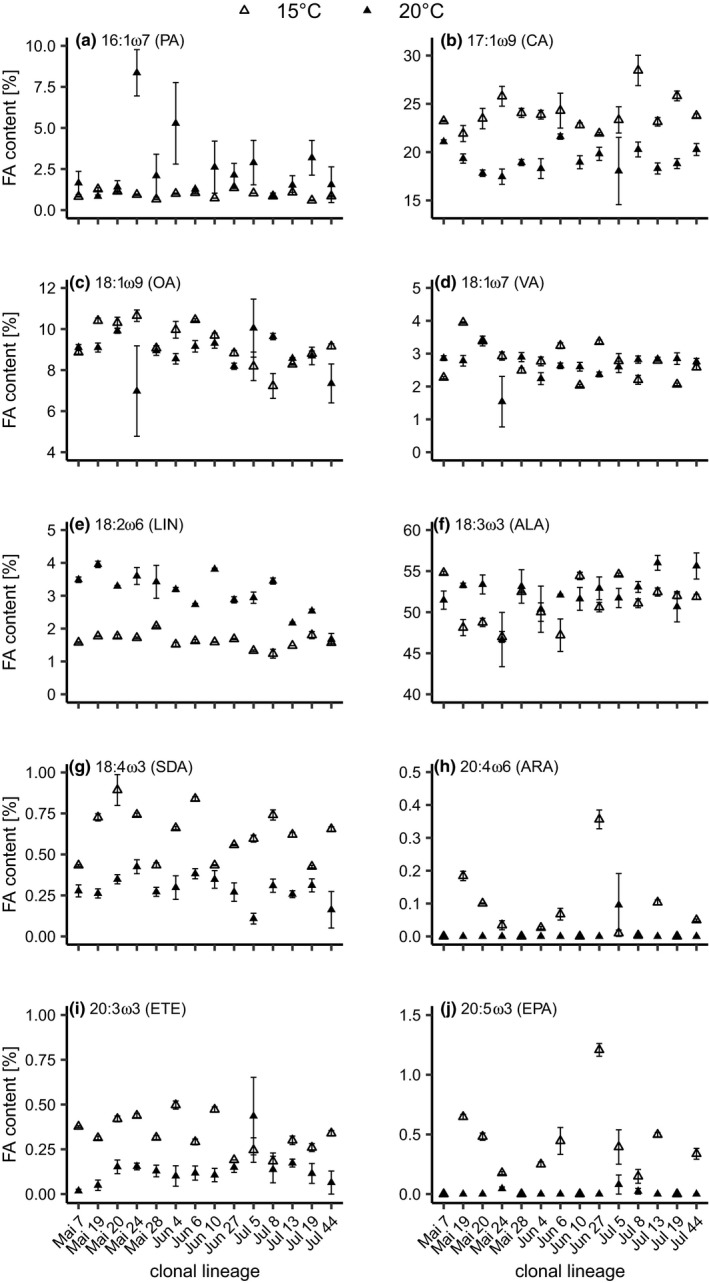
Mean (± SE) relative contents of a) palmitoleic acid (16:1ω7, PA), b) civetic acid (17:1ω9, CA), c) oleic acid (18:1ω9, OA), d) vaccenic acid (18:1ω7, VA), e) linoleic acid (LIN), f) α‐linolenic acid (ALA), g) stearidonic acid (SDA), h) arachidonic acid (ARA), i) eicosatrienoic acid (ETE), and j) eicosapentaenoic acid (EPA) of 14 *D. magna* clonal lineages isolated from Lake Bysjön grown at 15 and 20°C. *N* = 3; except for clonal lineages Mai 19, Jun 6, Jun 27, Jul 5, and Jul 13 at 15°C (*n* = 2). Note different scaling of y‐axis

The relative fatty acid (FA) content was determined in each clonal lineage at both temperatures. FA contents were calculated for palmitoleic acid (16:1ω7, PA), civetic acid (17:1ω9, CA), oleic acid (18:1ω9, OA), vaccenic acid (18:1ω7, VA), linoleic acid (18:2ω6, LIN), α‐linolenic acid (18:3ω3, ALA), stearidonic acid (18:4ω3, SDA), arachidonic acid (20:4ω6, ARA), eicosatrienoic acid (20:3ω3, ETE), and eicosapentaenoic acid (20:5ω3, EPA). We applied linear regression models analyzing the effect of *Temperature* and *Genotype* and the interaction of these parameters on the relative fatty acid content of each individual fatty acid. These linear regression models revealed significant differences of the main effect *Genotype* on all fatty acids except PA (16:1ω7), OA (18:1ω9), and ETE (20:3ω3) and of the main effect *Temperature* on all fatty acids except OA (18:1ω9) and VA (18:1ω7) (Figure 2a‐j; Table 1). Additionally, significant differences in the interaction between genotype and temperature on all fatty acids except EPA (20:5ω3) and ARA (20:4ω6) were revealed (Figure 2a‐j; Table 1).

In order to investigate if genotypes’ differences in the content of single unsaturated fatty acids are related to the observed differences in growth at the respective temperature, we tested for linear correlations of the relative FA data with juvenile growth rates. Mean juvenile growth rates did not show a significant correlation with any single fatty acid of the clonal lineages at either temperature (Table S1).

### 3.2 Genotype‐specific response patterns to changes in temperature

To further explore the evidence that clonal lineages utilize fatty acid unsaturation to different extent in order to adjust membrane fluidity as a reaction to changing temperatures, we calculated reaction norm slopes of the relative content of individual fatty acids between 15°C and 20°C for each of the genotypes. Hence, the slopes represent changes of the relative content of fatty acids between 15°C and 20°C, and we compared the slopes of each fatty acid among the genotypes by applying a linear regression model. When looking at individual fatty acids, we found that changes were significantly different among the genotypes (linear regression, Figure S1, Tables S2 and S3). These genotype‐specific differences in the profile of fatty acid content changes support the reasoning that genotypes may have different strategies to cope with changing temperatures.

**FIGURE 3:**
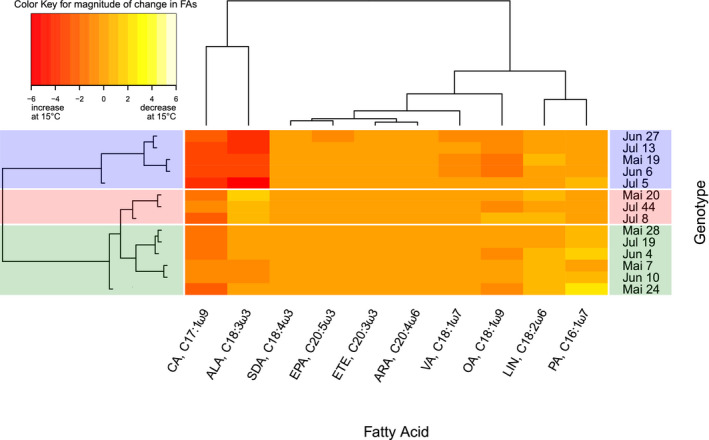
Hierarchical clustering of 14 genotypes according to the direction and magnitude of changes in the relative content of unsaturated fatty acids between 15°C and 20°C visualized in a heatmap. Red bars indicate an increase of the relative fatty acid content at 15°C. Yellow bars indicate a decrease of the relative fatty acid content at 15°C (and thus an increase at 20°C). Three clusters of genotypes generated by the hierarchical clustering analysis are shown in blue, red, and green boxes. Note: Negative slope values in the Color Key mean increase at 15°C

To disentangle the different patterns of genotypes in response to temperature changes, we applied a hierarchical clustering analysis that grouped genotypes according to differences in the direction and magnitude of changes in relative fatty acid content for each fatty acid plotted as a heatmap (Figure 3). The genotypes Mai 19, Jun 6, Jun 27, Jul 5, and Jul 13 clustered together and were separated from the other genotypes due to a stronger relative increase in CA (17:1ω9) and ALA (18:3ω3) at 15°C compared to the other genotypes (Figure 3, blue box). Mai 19, Jun 6, and Jun 27 additionally showed a stronger relative increase in the C18‐monounsaturated fatty acids (MUFAs) OA (18:1ω9) and VA (18:1ω7) compared to other genotypes. A second cluster of clonal lineages comprises the genotypes Mai 20, Jul 8, and Jul 44, in which the strongest decrease in relative ALA content at 15°C compared to 20°C was detected, separating this group from all other genotypes (Figure 3, red box). Genotypes Mai 7, Mai 24, Mai 28, Jun 4, Jun 10, and Jul 19 form a third cluster, separated from the other two clusters by only showing insignificant changes (both increase and decrease depending on the genotype) in the relative content of ALA and a decrease of PA (16:1ω7) at 15°C (Figure 3, green box). The relative content of the PUFAs SDA, ARA, ETE, and EPA increased at 15°C, but this increase did not differ among the genotypes, while that of LIN decreased for only some of the genotypes at 15°C.

**FIGURE 4:**
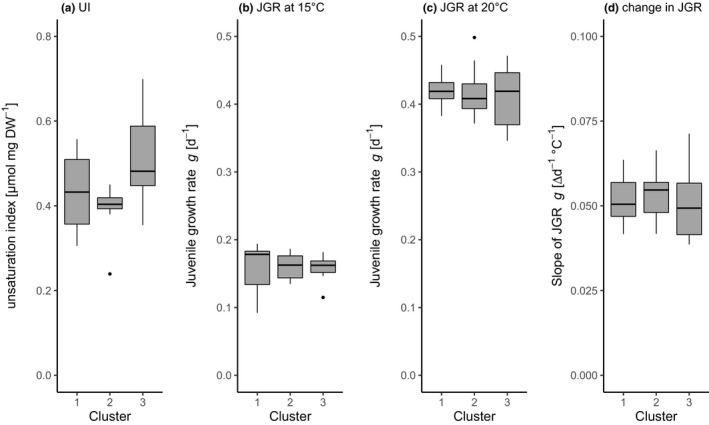
(a) Unsaturation index (UI) at 15°C, (b) JGR at 15°C, (c) JGR at 20°C and (d) change in JGR between 15°C and 20°C shown as reaction norm slopes of the three clusters of *D. magna* clonal lineages that resulted from the hierarchical clustering analysis (see Figure 3). Boxplots show median, first and third quartile (± SD). Clonal lineages were isolated from Lake Bysjön and grown at 15°C and 20°C, respectively. Cluster 1 (blue box in Figure 3): *N* = 5; Cluster 2 (red box in Figure 3): *N* = 3; Cluster 3 (green box in Figure 3): *N* = 6

To characterize the degree of fatty acid unsaturation at 15°C in the different genotypes, we calculated an unsaturation index (UI) based on fatty acid desaturation related to animal biomass according to Martin‐Creuzburg et al. (2019). This unsaturation index at 15°C did not differ among the three clusters of genotypes generated by the clustering analysis (Figure 4a; one‐way ANOVA, *F*_2_ = 2.423, *p* = .13). It is therefore reasonable to assume that these three clusters of genotypes increase the degree of desaturation in their membranes to the same extent, but they do so by different strategies, i.e. by altering the relative amount of different fatty acids. Therefore, we tested whether these different strategies also lead to different performances in terms of juvenile growth (JGR) of the three clusters of genotypes. A one‐way ANOVA showed neither differences for JGR at 15°C or 20°C nor for the change in JGR between the two temperatures (Figure 4b‐d; JGR 15°C: one‐way ANOVA, *F*_2_ = 0.11, *p* = .897; JGR 20°C: one‐way ANOVA, *F*_2_ = 0.288, *p* = .755; change in JGR: one‐way ANOVA, *F*_2_ = 0.091, *p* = .913). We conclude that the different strategies for achieving similar UIs at 15°C were not associated with differences in growth at either temperature.

## 4 DISCUSSION

### 4.1 Intrapopulation variation in temperature‐dependent growth and fatty acid content of 14 *D. magna* genotypes

The evolutionary capacity of aquatic populations to adapt to changes in environmental parameters such as temperature requires trait variation in the population in question (Geerts et al., 2015; Schlüter et al., 2014; Werner et al., 2019). Although it has been demonstrated for single *Daphnia* genotypes that they can adapt to different temperatures through a high degree of phenotypic plasticity, for example, physiological acclimation (e.g., Coggins et al., 2017; Pajk et al., 2012; Yampolsky et al., 2014; Zeis et al., 2019), an in‐depth study of genotype‐specific intrapopulation variation in responses to changing temperatures has to the best of our knowledge not yet been pursued. Here, we report significant temperature‐dependent variation in juvenile growth rate and relative fatty acid body content in a single *D. magna* population that was sampled from May to July 2010. We found significant differences in patterns of juvenile growth rate and fatty acids in 14 investigated genotypes within 15°C and 20°C but also between both temperatures. This is the first time that temperature‐dependent variation in juvenile growth rate and fatty acid patterns has been assessed in a natural *Daphnia* population.

Population dynamics within and among *Daphnia* species have been frequently studied. Most of these studies addressed producer–consumer (food quality and sensitivity to toxicity), host–parasite (survival and fecundity), and predator–prey (e.g., predator‐mediated changes in prey life history) systems. In the presence/absence of fish kairomones (simulating the presence/absence of predators), intraspecific variation in life‐history traits such as size or age at maturity and habitat use was demonstrated among *D. magna* populations (Boersma et al., 1998) as well as among *D. hyalina* × galeata hybrid clones (De Meester & Weider, 1999) and among populations of *D. galeata* (Tams et al., 2018). Furthermore, clonal variation in sensitivity to the toxic cyanobacterium *Microcystis* sp. of *D. pulicaria* and *D. pulex* genotypes was reported (Chislock et al., 2013; Hietala et al., 1997; Jiang et al., 2013, 2015). With respect to nutrient stoichiometry, interclonal variation has been shown for *D. pulex* in their response to varying food quality (Weider et al., 2005). Similarly, a previous study has shown that dietary availability of fatty acids, particularly EPA (eicosapentaenoic acid, 20:5ω3), leads to interclonal differences in eight *D. hyalina* clones, eight clones of the *D. hyaline* × *galeata* complex, and two *D. galeata* clones (Brzeziński et al., 2010; Brzeziński & von Elert, 2007). However, all of these previous studies did not use coexisting genotypes of a natural *Daphnia* population from the same lake, but used clones with temporal (time point of isolation) and spatial (habitat) differences in their origin. Here, we examined variations in juvenile growth rate and fatty acid patterns in a natural *Daphnia* population of coexisting genotypes from the same lake and thus with a common evolutionary history. In a recent paper, we reported genotype‐specific variation in heat tolerance within this very population (Werner et al., 2019). In line with this, Ilić et al. (2021) demonstrated intrapopulation variation in juvenile growth rate and the susceptibility to limitations in dietary fatty acid availability of a natural *D. longispina* population at 20°C.

In order to test if the observed variation in juvenile growth rate within the respective temperatures might be explained by the variation observed in single fatty acids, we correlated juvenile growth rate with all single fatty acids at either temperature, but none of the correlations were significant.

### 4.2 Genotype‐specific adjustment of fatty acid composition to changes in temperature

Variation in fatty acid patterns between 15°C and 20°C may be explained by the concept of homeoviscous adaptation. In response to changes in ambient temperature, *Daphnia* attempt to adjust their membrane lipid composition to maintain proper membrane fluidity and function (Hazel, 1995; Hazel & Williams, 1990). In general, the fluidity of a given membrane is reduced at decreasing ambient temperatures, whereas it is increased at increasing temperatures (Hazel, 1995). The adjustment of membrane lipid composition by *Daphnia* and other invertebrates involves the remodeling of membrane lipids by altering the chain length and the degree of unsaturation of fatty acids (Guschina & Harwood, 2006). In response to lower ambient temperatures, increased levels of polyunsaturated fatty acids (PUFAs) in the membrane of *Daphnia* and thus increased membrane fluidity are expected (Farkas, 1979; Hazel, 1995).

In line with several other studies, we have determined fatty acid changes in body tissue and investigated if these changes are in accordance with expectations of homeoviscous adaptation (Hahn & von Elert, 2020; Schlechtriem et al., 2006; Sperfeld & Wacker, 2012; Werner et al., 2019; Zeis et al., 2019). Extending these aforementioned studies, we have in addition used these changes in body tissue fatty acids to calculate an unsaturation index of membranes in order to infer effects on membrane fluidity and thus homeoviscous adaptation as according to Martin‐Creuzburg et al. (2019). In earlier papers, increased concentrations of ω3‐PUFAs (Sperfeld & Wacker, 2012; Zeis et al., 2019) and total PUFAs (von Elert & Fink, 2018) in body tissue of *D. magna* at 15°C compared to 20°C were reported, indicating that the body PUFA content of *Daphnia* increases at lower ambient temperatures, which is consistent with the concept of homeoviscous adaptation. It should be mentioned, however, that neither membrane fluidity nor membrane fatty acids but instead body fatty acids have been analyzed in these studies, and thus, the concept of homeoviscous adaptation was not tested specifically. The demand for increased concentrations of ω3‐PUFAs at lower temperatures has been observed in particular for EPA. In supplementation experiments at 15°C and 20°C, a higher EPA requirement for growth at the lower temperature has been observed (Sperfeld & Wacker, 2011). However, it has also been suggested that growth of *D. magna* at 10°C compared to higher temperatures may not be limited by the low dietary availability of a particular PUFA, but by the low availability of PUFAs in general (Martin‐Creuzburg et al., 2012). In terms of homeoviscous adaptation, these evidences for increased PUFA requirement at lower temperatures can be assigned to the highly bent carbon chains of PUFAs in general as a result of a higher degree of unsaturation (increased number of double bonds) in the membrane and thus reduced van der Waals interactions between membrane fatty acids (Murray et al., 2010). These reduced van der Waals interactions between membrane fatty acids lead to a lower melting point of the membrane and thus increase the fluidity of the membrane by containing more unsaturated fatty acids, so that it is not a particular PUFA but rather the overall degree of unsaturation in cell membranes that affects membrane fluidity. Constraints in the fitness of *Daphnia* at low temperatures may therefore most likely be caused by suboptimally adjusted membrane fluidity as a consequence of a general PUFA limitation rather than of a limitation by specific PUFAs.

In the 14 genotypes of the population investigated in our study, the body content of the ω3‐PUFAs SDA (stearidonic acid, 18:4ω3), ETE (eicosatrienoic acid, 20:3ω3), and EPA and the ω6‐PUFA ARA (arachidonic acid, 20:4ω6) increased at 15°C compared to 20°C, which is in accordance with results shown for a single clone by Sperfeld and Wacker (2012), while the body content of monounsaturated fatty acids (MUFAs), of the ω3‐PUFA ALA (α‐linolenic acid, 18:3ω3) and of the ω6‐PUFA LIN (linoleic acid, 18:2ω6) in contrast to that study did not increase at the lower temperature across all genotypes in our experiments, but remained unaffected (overall MUFAs and ALA) or even decreased (LIN). In a recent paper by Zeis et al. (2019), the concentrations of all ω3‐PUFAs (ALA, SDA, ETE, EPA) and also of the ω6‐PUFA LIN increased in *D. magna* cultivated at a lower acclimation temperature (10°C vs. 20°C) and only that of ARA decreased at a lower acclimation temperature. Hence, though Sperfeld and Wacker (2012) and Zeis et al. (2019) investigated *D. magna*, both studies differ with respect to which individual fatty acids increased or decreased in response to decreasing temperatures. These discrepancies can most likely be explained by the fact that in both studies only a single genotype was tested, which can never reflect the variation within a population. In our study of 14 genotypes in a population, we observed genotype‐specific changes in both directions (both increase and decrease) for individual fatty acids (e.g., ALA) in response to lower temperature. As an example may be taken ALA, which is by far the most abundant PUFA in *D. magna* in all three studies discussed here: While ALA increased at 10°C and 15°C compared to 20°C in Zeis et al. (2019) and in Sperfeld and Wacker (2012), respectively, we here find this response in 9 out of 14 clones, whereas 5 clones responded with a decrease in ALA. The different observations of both Sperfeld and Wacker (2012) and Zeis et al. (2019) therefore apply to some of our genotypes but not to all. The probability that these two studies would also have found genotype‐specific results if they had tested multiple genotypes from the same population is high, in our opinion. However, it should also be mentioned that we used relative fatty acid concentrations in our study, whereas absolute fatty acid concentrations were used in the other two studies, and that a slight increase in absolute fatty acid concentration does not necessarily lead to an increase in the relative concentration of the respective fatty acid.

Nevertheless, increased incorporation of PUFAs that exhibit a high degree of unsaturation (e.g., SDA, ARA, ETE, EPA; all at least 3 or more double bonds), at 15°C is consistent with the concept of homeoviscous adaptation and the goal of maintaining membrane fluidity at a lower temperature. We tested for genotype‐specific changes in the relative content of unsaturated fatty acids upon exposure to 20°C and 15°C by applying a linear model to the slopes of reaction norms between the two temperatures, and we hypothesized to find genotype‐specific changes in fatty acid composition. Here, we report for the first time significant variation in the changes of the composition of unsaturated fatty acids upon exposure to 15°C and 20°C in a natural *D. magna* population. These genotype‐specific profiles of changes in the composition of unsaturated fatty acids hint at genotype‐specific strategies to cope with changing temperatures.

By means of hierarchical clustering, we identified three clusters of genotypes that upon exposure to the lower temperature changed their relative content of different fatty acids and thus reacted by pursuing different strategies to cope with the change of ambient temperature. Here, we calculated a so‐called unsaturation index (UI) as a proxy of membrane fluidity to test if these different strategies in fatty acid alteration were also reflected in different levels of membrane fluidity of the three clusters of genotypes. Membrane fluidity, typically assessed by fluorescence polarization, has been shown to correlate well with increasing ratios of unsaturated fatty acids to saturated fatty acids (UFA/SFA; Cossins & Prosser, 1978; Hazel & Williams, 1990) and with the unsaturation index (UI; Cossins & Prosser, 1978). This UI has been calculated in various ways in recent papers with *D. magna*. Hahn et al. (2020) calculated an UI following Cossins and Prosser (1978) by relating the number of double bonds to total fatty acids including saturated fatty acids (SFAs), which are assumed to negatively affect membrane fluidity. In Martin‐Creuzburg et al. (2019), an UI was calculated based on fatty acid desaturation related to animal biomass, not considering the concentration of SFAs. Additionally, in that study, membrane fluidity in the experimental animals was determined by fluorescence polarization and shown to be well correlated with the calculated UI (Martin‐Creuzburg et al., 2019). Since this UI was shown to be related to membrane fluidity (determined by fluorescence polarization), we here calculated the UI according to Martin‐Creuzburg et al. (2019). For the same reason, we followed Martin‐Creuzburg et al. (2019) and used fatty acid analyses of whole body extracts of *Daphnia* for the calculation of the UI and regarded the UI as a proxy for membrane fluidity at 15°C, a temperature at which *Daphnia* are expected to increase the share of PUFAs in their cell membranes. However, we have not assessed membrane fluidity itself.

We hypothesized that genotype‐specific changes in the fatty acid composition of a *D. magna* population would translate into different UI values and thus different membrane fluidity of genotypes upon exposure to 15°C, which would suggest that clonal lineages rely to different extent on fatty acid unsaturation to adjust homeoviscosity of their membrane. However, the calculated UI values did not differ between the three generated clusters of genotypes. Therefore, we conclude that genotypes adjust the fluidity of their membranes by following different strategies in terms of changing the composition of different fatty acids in their membranes, but these different strategies do not result in differences in membrane fluidity.

The three clusters of genotypes were mainly separated by the magnitude and direction of change in ALA, but also changes in CA (civetic acid, 17:1ω9), OA (oleic acid, 18:1ω9), and VA (vaccenic acid, 18:1ω7) contributed to the clustering, while SDA, ARA, ETE, and EPA, although present in increased shares at 15°C, did not differ in the magnitude of this increase between genotypes. Differently increased ALA levels in the three clusters of genotypes may also be reflected in different fitness levels of these clusters, as ALA has previously been associated with fitness in *Daphnia* in numerous studies (von Elert, 2002; Wacker & von Elert, 2001; Windisch & Fink, 2018). However, the fitness parameter assessed in our study, juvenile growth rate, did not differ between the three clusters.

### 4.3 Conclusion

We found that clusters of genotypes of a single *D. magna* population increased the degree of unsaturation (UI) in their body at a lower temperature to the same extent, but achieve this by pursuing different strategies in terms of altering the relative share of different fatty acids. We also found that these different strategies of responding to changes in temperature did not influence fitness measured as juvenile growth rate. Hence, though the clusters of *Daphnia* genotypes pursue different strategies, their phenotypes converge with respect to UI and juvenile growth rate. The different genotype clusters represent different physiological solutions, and, as PUFA‐limitation is widespread in nature, these genotypes will be subjected to different degrees of PUFA‐limitation when sharing the same diet, which may render some of them temporarily competitively superior. However, as PUFA availability changes seasonally with phytoplankton, this will result in temporal fluctuations of competitive superiority among the genotypes and lead only to short‐term microevolutionary changes in the *Daphnia* population. It has been suggested that there is no long‐term directional selection as phytoplankton succession is repeated every year (Schaffner et al., 2019), which well explains why we find this pronounced standing variation in utilization of different PUFAs within the population, which results in similar changes in unsaturation upon exposure to low temperatures.

## CONFLICT OF INTEREST

There is no conflict of interest.

## AUTHOR CONTRIBUTION

**Christian Werner:** Conceptualization (equal); Data curation (lead); Formal analysis (lead); Investigation (lead); Methodology (equal); Project administration (equal); Supervision (equal); Validation (equal); Visualization (lead); Writing‐original draft (lead); Writing‐review & editing (equal). **Kathrin A. Otte:** Data curation (supporting); Formal analysis (supporting); Methodology (supporting); Visualization (supporting); Writing‐review & editing (equal). **Eric von Elert:** Conceptualization (equal); Funding acquisition (lead); Methodology (equal); Project administration (equal); Resources (lead); Supervision (equal); Validation (equal); Writing‐review & editing (equal).

## Supporting information

Supinfo

Figure S1

## Data Availability

The data that support the findings of this study are openly available in Dryad at https://doi.org/10.5061/dryad.m905qfv21
