# Supplementary material for: Phenotypic convergence in a natural Daphnia population acclimated to low temperature
Source: Ecol Evol. 2021 Oct 12;11(21):15312–24. doi: 10.1002/ece3.8217 (PMC8571613; doi:10.1002/ece3.8217)
Supplement: Supplementary file 1 — Supinfo [file ECE3-11-15312-s002.docx]

### Supplementary Material


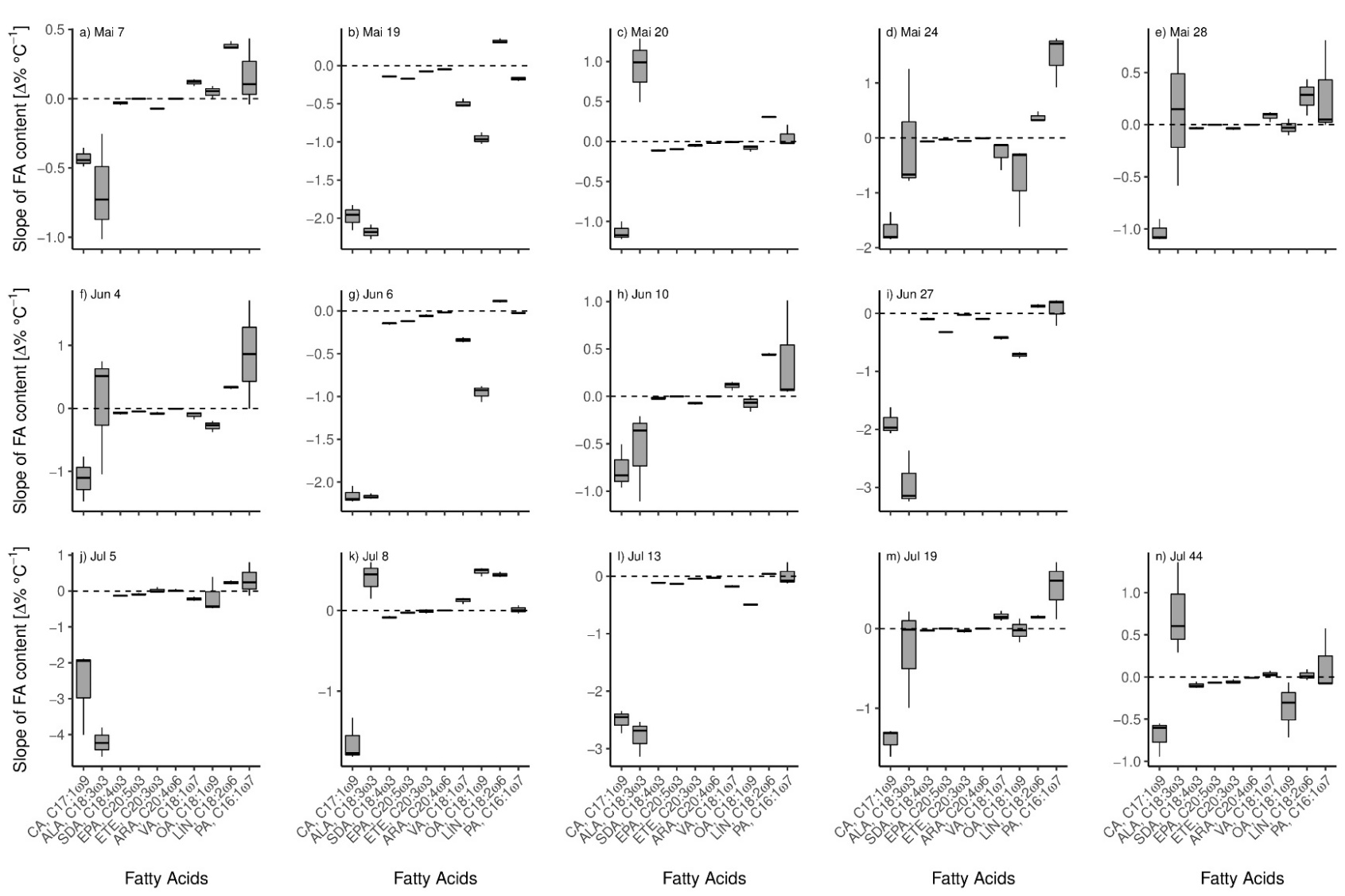


**Supplementary Fig. 1** Change in relative fatty acid content between 15°C and 20°C shown as reaction norm slopes of single fatty acids for each genotype (a-n). *N* = 3 for each fatty acid per genotype. Negative slopes/values mean increase at 15°C, positive slopes/values mean decrease at 15°C. Dashed horizontal line through zero represents no change between temperatures. Fatty acid order is the same as in the heatmap (Fig. 3). Note: Different scaling of y-axis.

**Supplementary Table 1** Linear Correlations of mean growth rate with mean fatty acid body content of each fatty acid at the respective temperature of 14 *D. magna* clonal lineages isolated from Lake Bysjön grown at 15°C and 20°C. P-values indicate no significant correlations after Benjamini-Hochberg (BH) correction.

| **Response variable** | **Fatty Acid** | ***Temperature*** | ***p*** |
| --- | --- | --- | --- |
| Juvenile growth rate | 16:1$\omega$7 (PA)  17:1$\omega9$ (CA)  18:1$\omega9$(OA)  18:1$\omega$7 (VA)  18:2$\omega6$ (LIN)  18:3$\omega3$ (ALA)  18:4$\omega3$ (SDA)  20:4$\omega6$ (ARA)  20:3$\omega3$ (ETE)  20:5$\omega3$ (EPA) | 20°C 20°C 20°C  20°C  20°C  20°C  20°C  20°C  20°C  20°C | 0.966  0.977  0.966  0.504  0.966  0.966  0.966  0.966  0.971  0.966 |
| Juvenile growth rate | 16:1$\omega$7 (PA)  17:1$\omega9$ (CA)  18:1$\omega9$(OA)  18:1$\omega$7 (VA)  18:2$\omega6$ (LIN)  18:3$\omega3$ (ALA)  18:4$\omega3$ (SDA)  20:4$\omega6$ (ARA)  20:3$\omega3$ (ETE)  20:5$\omega3$ (EPA) | 15°C 15°C 15°C  15°C  15°C  15°C  15°C  15°C  15°C  15°C | 0.966  0.966  0.966  0.971  0.966  0.966  0.966  0.966  0.9660.966 |

**Supplementary Table 2** Results from the linear regression model showing effects of the factors Genotype, Fatty Acid, and their interaction on reaction norm slopes that represent changes of the relative content of fatty acids between 15°C and 20°C of the investigated *Daphnia magna* population (all 14 genotypes included). P-values in bold indicate significant effects.

| **Response variable** | **Effect** | ***df*** | ***F*** | ***p*** |
| --- | --- | --- | --- | --- |
| Reaction norm slope | Genotype Fatty Acid Genotype × Fatty Acid | 13 9 117 | 16.87 91.84 5.87 | **<0.001 <0.001 <0.001** |

**Supplementary Table 3** Pairwise comparisons of all 14 genotypes per fatty acid following the linear regression model showing effects of the factors *Genotype*, *Fatty* *Acid*, and their interaction on reaction norm slopes that represent changes of the relative content of fatty acids between 15°C and 20°C of the investigated *Daphnia magna* population (Supplementary Table 2). Significant P-values after Tukey correction lead to different profiles of genotypes regarding the change of fatty acid content between 15°C and 20°C. Only significant comparisons are shown due to the high number of comparisons (>900).

| **Fatty Acid** | **Contrast** | ***p*** |
| --- | --- | --- |
| 16:1$\omega$7 (PA) | Mai 19 – Mai 24  Mai 20 – Mai 24  Mai 24 – Mai 7  Mai 24 – Mai 28  Jun 4 – Mai 7  Jun 4 – Mai 19  Jun 4 – Mai 20  Jun 4 – Mai 24  Jun 4 – Mai 28  Jun 4 – Jun 6  Jun 6 – Mai 24  Jun 10 – Mai 24  Jun 27 – Mai 24  Jun 27 – Jun 4  Jul 5 – Mai 24  Jul 5 – Jun 4  Jul 8 – Mai 24  Jul 8 – Jun 4  Jul 13 – Mai 24  Jul 13 – Jun 4  Jul 19 – Mai 19  Jul 19 – Mai 24  Jul 44 – Mai 24  Jul 44 – Jun 4 | **<0.001<0.001<0.001<0.001**  **<0.001**  **<0.001**  **<0.001**  **0.002 0.01 <0.001<0.001<0.001<0.001<0.001<0.0010.02 <0.001<0.001<0.001<0.0010.02 <0.001<0.001<0.001** |
| 17:1$\omega9$ (CA) | Mai 19 – Mai 7  Mai 24 – Mai 7  Jun 6 – Mai 7  Jun 27 – Mai 7  Jul 5 – Mai 7  Jul 5 – Jun 10  Jul 8 – Mai 7  Jul 13 – Mai 7  Jul 13 – Jun 10  Jul 13 – Jul 44  Jul 44 – Jun 6  Jul 44 – Jul 5 | **0.003 0.025 <0.0010.006 <0.0010.04 0.03 <0.001 0.017 0.007 0.034 0.019** |
| 18:1$\omega9$(OA) | Mai 19 – Mai 7  Mai 19 – Mai 28  Jun 6 – Mai 7  Jun 6 – Mai 28  Jul 8 – Mai 19  Jul 8 – Mai 24  Jul 8 – Jun 4  Jul 8 – Jun 6  Jul 8 – Jun 27  Jul 13 – Jul 8  Jul 19 – Mai 19  Jul 19 – Jun 6  Jul 44 – Jul 8 | **0.008 0.026 0.008 0.027 <0.001<0.001 0.013 <0.001<0.001<0.0010.025 0.025 0.005** |
| 18:3$\omega3$ (ALA) | Mai 19 – Mai 7  Mai 19 – Mai 20  Mai 19 – Mai 24  Mai 19 – Mai 28  Mai 20 – Mai 7  Mai 20 – Mai 24  Mai 20 – Mai 28  Mai 28 – Mai 7  Jun 4 – Mai 7  Jun 4 – Mai 19  Jun 4 – Mai 20  Jun 4 – Jun 6  Jun 6 – Mai 7  Jun 6 – Mai 20  Jun 6 – Mai 24  Jun 6 – Mai 28  Jun 10 – Mai 19  Jun 10 – Mai 20  Jun 10 – Jun 6  Jun 10 – Jun 27  Jun 27 – Mai 7  Jun 27 – Mai 20  Jun 27 – Mai 24  Jun 27 – Mai 28  Jun 27 – Jun 4  Jul 5 – Mai 7  Jul 5 – Mai 20  Jul 5 – Mai 24  Jul 5 – Mai 28  Jul 5 – Jun 4  Jul 5 – Jun 10  Jul 5 – Jul 8  Jul 8 – Mai 7  Jul 8 – Mai 19  Jul 8 – Jun 6  Jul 8 – Jun 10  Jul 8 – Jun 27  Jul 13 – Mai 7  Jul 13 – Mai 20  Jul 13 – Mai 24  Jul 13 – Mai 28  Jul 13 – Jun 4  Jul 13 – Jun 10  Jul 13 – Jul 8  Jul 13 – Jul 19  Jul 13 – Jul 44  Jul 19 – Mai 19  Jul 19 – Mai 20  Jul 19 – Jun 6  Jul 19 – Jun 27  Jul 19 – Jul 5  Jul 19 – Jul 44  Jul 44 – Mai 7  Jul 44 – Mai 19  Jul 44 – Mai 24  Jul 44 – Mai 28  Jul 44 – Jun 4  Jul 44 – Jun 6  Jul 44 – Jun 10  Jul 44 – Jun 27  Jul 44 – Jul 5 | **0.017 <0.001<0.001<0.001 <0.001<0.001<0.0010.022 0.024 <0.001<0.001 <0.0010.018 <0.001<0.001<0.001 0.003 <0.0010.004 <0.0010.001 <0.001<0.001<0.001 <0.001<0.001<0.001<0.001<0.001<0.001<0.001<0.001<0.001<0.001<0.0010.002 <0.0010.001 <0.001<0.001<0.001<0.001<0.001<0.001<0.001<0.001 <0.001<0.001<0.001<0.001<0.001<0.001<0.001<0.0010.012 0.043 0.039 <0.001<0.001<0.001<0.001** |
