# Supplementary figures and images for: Phenotypic convergence in a natural Daphnia population acclimated to low temperature
Source: Ecol Evol. 2021 Oct 12;11(21):15312–24. doi: 10.1002/ece3.8217 (PMC8571613; doi:10.1002/ece3.8217)

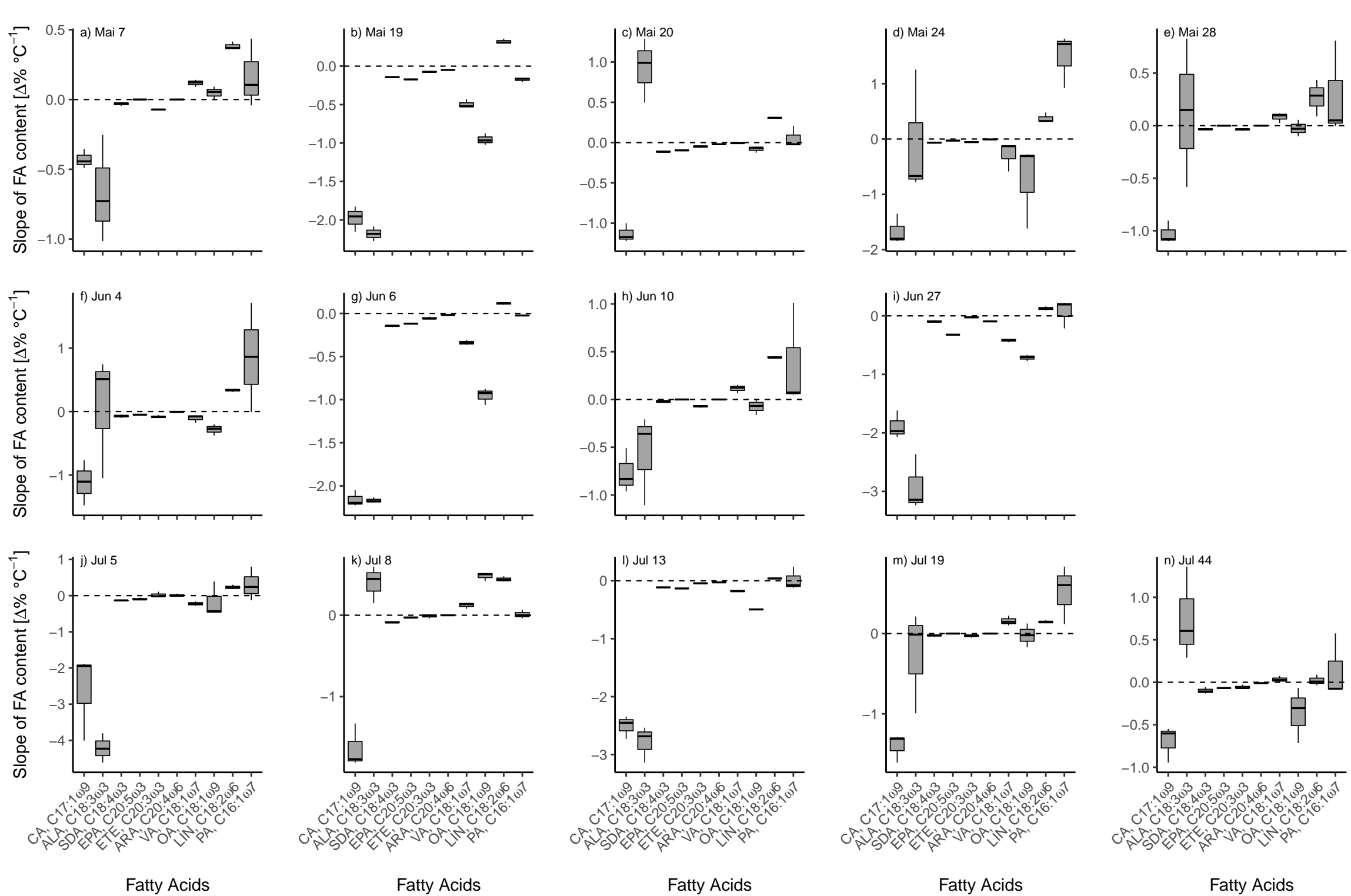

Supplement: Supplementary file 2 — Figure S1 [file ECE3-11-15312-s001.pdf]
